# Safety and immune response kinetics of GRAd-COV2 vaccine: phase 1 clinical trial results

**DOI:** 10.1038/s41541-022-00531-8

**Published:** 2022-09-24

**Authors:** Chiara Agrati, Concetta Castilletti, Simone Battella, Eleonora Cimini, Giulia Matusali, Andrea Sommella, Alessandra Sacchi, Francesca Colavita, Alessandra M. Contino, Veronica Bordoni, Silvia Meschi, Giulia Gramigna, Federica Barra, Germana Grassi, Licia Bordi, Daniele Lapa, Stefania Notari, Rita Casetti, Aurora Bettini, Massimo Francalancia, Federica Ciufoli, Alessandra Vergori, Serena Vita, Michela Gentile, Angelo Raggioli, Maria M. Plazzi, Antonella Bacchieri, Emanuele Nicastri, Andrea Antinori, Stefano Milleri, Simone Lanini, Stefano Colloca, Enrico Girardi, Roberto Camerini, Giuseppe Ippolito, Francesco Vaia, Antonella Folgori, Stefania Capone

**Affiliations:** 1 Istituto Nazionale per Le Malattie Infettive Lazzaro Spallanzani IRCCS, Rome, Italy; 2 ReiThera Srl, Rome, Italy; 3 Clinical Research and Development Consultants srls, Sesto Fiorentino, Florence, Italy; 4 Centro Ricerche Cliniche di Verona srl, Verona, Italy

**Keywords:** Live attenuated vaccines, Viral infection, Adaptive immunity

## Abstract

Despite the successful deployment of efficacious vaccines and therapeutics, the development of novel vaccines for SARS-CoV-2 remains a major goal to increase vaccine doses availability and accessibility for lower income setting. We report here on the kinetics of Spike-specific humoral and T-cell response in young and old volunteers over 6 months follow-up after a single intramuscular administration of GRAd-COV2, a gorilla adenoviral vector-based vaccine candidate currently in phase-2 of clinical development. At all three tested vaccine dosages, Spike binding and neutralizing antibodies were induced and substantially maintained up to 3 months, to then contract at 6 months. Potent T-cell responses were readily induced and sustained throughout the study period, with only minor decline. No major differences in immune response to GRAd-COV2 vaccination were observed in the two age cohorts. In light of its favorable safety and immunogenicity, GRAd-COV2 is a valuable candidate for further clinical development and potential addition to the COVID-19 vaccine toolbox to help fighting SARS-CoV-2 pandemic.

## Introduction

SARS-CoV-2 pandemic has caused unprecedented major disruptions to public health, economy and social life, with a tragical outcome of more than 5.8 million lives lost globally as of February 2022^1^. The race for developing effective vaccines has been fast and successful, thanks to a coordinated effort of academic and private biotechnology as well as pharmaceutical sectors. Unprecedented and timely public investments were also instrumental for this achievement. Amongst the different traditional and innovative vaccine technologies that where challenged, two genetic vaccine approaches emerged and delivered most rapidly the first wave of effective vaccines approved for emergency use, namely the mRNA and the adenoviral vector technologies^2–4^.

The fast development speed is a hallmark of these two technologies, since they both rely on the quick adaptation of the nucleic acid cargo (either RNA or DNA) to the antigen of interest but with essentially unaffected manufacturing, purification and formulation processes; these features led already in late 2020 to the demonstration of efficacy^5–8^ and approval of the first vaccines for COVID-19, Pfizer/Biontech (BNT162b2), Moderna (mRNA-1273), Oxford-AstraZeneca (ChAdOx1 nCoV-19/AZD1222), soon followed by Janssen/Johnson & Johnson (Ad26.COV2.S). Notably, this was achieved less than 12 months after the Wuhan SARS-CoV-2 Spike protein coding sequence was made available to the scientific community. In 2021, up to 33 vaccines based on different technologies were authorized or fully approved at least in one country^9^ and showed various levels of efficacy and effectiveness against symptomatic infection^10–17^, but all provided some degree of cross-neutralizing antibodies to the periodically emerging variants of concern (VOC)^18–21^ and substantial protection from severe disease. However, the evidence of waning humoral immunity within 4–6 months from primary vaccination series^22–30^ has raised a debate on the need to adapt current or novel vaccines to the circulating strain and has set the ground for the worldwide adoption of booster vaccinations to efficiently counteract circulating VOCs.

From a mechanism of action perspective, genetic vaccines can model the host-pathogen interaction better than either inactivated or subunit vaccines, since the encoded immunogenic antigens are produced intracellularly by host cells. This feature in turn allows MHC class I epitope presentation for optimal induction of CD8 responses along with Th1 polarization^31^. Adenoviral vectors, originally exploited as gene therapy vehicles, soon revealed to be potent genetic vaccine carriers. They have since been widely employed to develop candidate vaccines to a variety of infectious agents and cancers^32,33^. Despite decades of clinical development, before the COVID-19 era only three preventive vaccines based on the adenoviral vector platform for a single indication (Ebola) were recently approved^34,35^. The adenoviral vector platform is well recognized to induce antibodies, and even more T-cell responses with a strong CD8 component to the encoded transgene for which this technology is considered best in class^36^, in animal models and in a wide variety of clinical trials and real life observational studies^31^. Other favorable characteristics, such as the possibility to manufacture at scale at reasonable dose cost and storage at refrigerator temperature pose the adenoviral platform at the forefront of promising approaches to combat infectious diseases, especially in low-middle income countries (LMIC) context. The recognition of very rare but serious syndrome, named vaccine-induced thrombosis with thrombocytopenia (VITT), occurring 4–30 days after receiving the first dose of ChAdOx1 nCov-19^37^ and Ad26.COV2.S^38^ represented a drawback for the technology, that led many high-income countries to adopt careful risk/benefit analysis approaches or age restrictions in their continued usage or to abandon their implementation in national vaccination campaigns. Despite this very rare adverse event whose mechanism is under extensive investigation, adenoviral vectored COVID-19 vaccines are still at the basis of national campaigns in less developed countries. Indeed, the emergence of omicron prompted a rush to the adoption of third and even fourth vaccine dose; considering that large sectors of the world population are not even yet fully covered by the primary vaccination cycle, adenovirus-based vaccines are regarded as an important option for heterologous primary or boosting schedules. The review of literature available^39–46^ suggest similar immunogenicity of heterologous regimens incorporating mRNA and adenoviral vectored vaccines compared to the homologous schedule, with acceptable safety profiles, as recognized in the recently issued (December 16, 2021) interim recommendations for heterologous COVID-19 vaccine schedules by the WHO Strategic Advisory Group of Experts (SAGE) on Immunization^47^. A flexible approach in heterologous schedule implementation is regarded as reasonable, taking into account practical considerations such as vaccine supply and availability or logistic issues, with the aim of reaching maximal vaccine coverage globally. Therefore, the continued effort for developing new adenoviral vectored COVID-19 vaccines is still supported, considering that differently from antibodies, T cell responses induced by Wuhan-based vaccines have been repeatedly shown to almost fully cross-recognize Spike CD4 and CD8 epitopes from all VOCs^48–51^, including omicron^52–54^.

We recently reported on the interim analysis of a phase 1 clinical trial (RT-CoV-2 study) investigating safety and immunogenicity of GRAd-COV2, a gorilla adenovirus-based candidate vaccine for COVID-19;^55^ we were able to show a favorable safety profile across the three doses tested and the induction of humoral and cellular responses to SARS-CoV-2 Spike in younger and older age volunteers up to 4 weeks post vaccination^48^. Here we report on the complete immunological and virological 6 months follow-up of the volunteers, with the inclusion of serology assays calibrated to the WHO international standard to express binding and neutralizing antibodies for better comparison with other vaccines.

## Results

### Study design and vaccine safety

The study was an open-label, dose-escalation phase 1 clinical trial (RT-CoV-2 study) enrolling healthy volunteers belonging to two cohorts based on age: younger adults (18 to 55 years old) and older adults (65–85 years old). Each cohort consisted of 3 arms of 15 volunteers each (a total of 6 study arms), for assessing a single intramuscular administration at three different doses of GRAd-COV2: low dose (L, 5 × 10^10^ viral particles, vp), intermediate dose (I, 1 × 10^11^ vp), and high dose (H, 2 × 10^11^ vp). The three study arms in each age cohort were well balanced for all demographic variables (Table 1), and overall male gender was prevalent in all groups (almost 2:1 M/F ratio). We previously reported on GRAd-COV2 safety and immunogenicity up to 1 month of follow up^48^ and we currently extend the observation period to 6 months. The vaccine was well tolerated, with no serious adverse events (SAE) or deaths reported (Supplementary Table 1). The adverse events (AEs) were mostly mild and moderate in grade, and resolved within few days, similar to other vaccines. The most frequent local event was injection site pain. Common systemic symptoms included fatigue, headache, pyrexia, chills and myalgia (ref. ^48^ and Supplementary Table 2).

**Table 1:** Study arms and volunteers characteristics.

Demographic Variable	Arm 1-LD (*N* = 15)	Arm 2-ID (*N* = 15)	Arm 3-HD (*N* = 15)	Y-Total (*N* = 45)	Arm 4-LD (*N* = 16)^1^	Arm 5-ID (*N* = 15)	Arm 6-HD (*N* = 15)	O-Total (*N* = 46)	All (*N* = 91)
Age (years)									
Mean	33.9	37.0	36.2	35.7	69.5	70.8	70.0	70.1	53.1
SD	9.1	10.2	11.1	10.1	4.6	5.6	3.5	4.6	18.9
Gender									
Male n (%)	8 (53)	10 (67)	9 (60)	27 (60)	11 (69)	10 (67)	10 (67)	31 (67)	58 (64)
Female n (%)	7 (47)	5 (33)	6 (40)	18 (40)	5 (31)	5 (33)	5 (33)	15 (33)	33 (36)
Weight (kg)									
Mean	73.9	69.7	70.9	71.5	73.6	70.4	70.9	71.7	71.6
SD	12.2	14.9	13.5	13.4	12.9	10.2	10.5	11.1	12.2
Height (cm)									
Mean	173.3	171.9	173.8	173.0	171.9	169.0	168.1	169.7	171.3
SD	9.8	10.9	10.2	10.1	8.1	9.0	11.3	9.5	9.9
BMI (kg/m^2^)									
Mean	24.5	23.4	23.3	23.7	24.8	24.6	25.0	24.8	24.3
SD	2.6	3.0	2.8	2.8	3.1	2.6	2.2	2.6	2.7

*N* number of subjects in the SAF, *SAF* Safety Analysis Set, *SD* standard deviation, *Y* Younger age volunteers, *O* older age volunteers, *LD* Low dose (5 × 10^10^ viral particles), *ID* Intermediate Dose (1 × 10^11^ viral particles), *HD* High Dose (2 × 10^11^ viral particles);^1^one subject in arm 4 was found seropositive to Spike at baseline and was substituted; the seropositive subject was followed up for vaccine safety but excluded from immunological analysis.

### Spike and receptor binding domain (RBD) specific binding antibody response

**Fig. 1:**
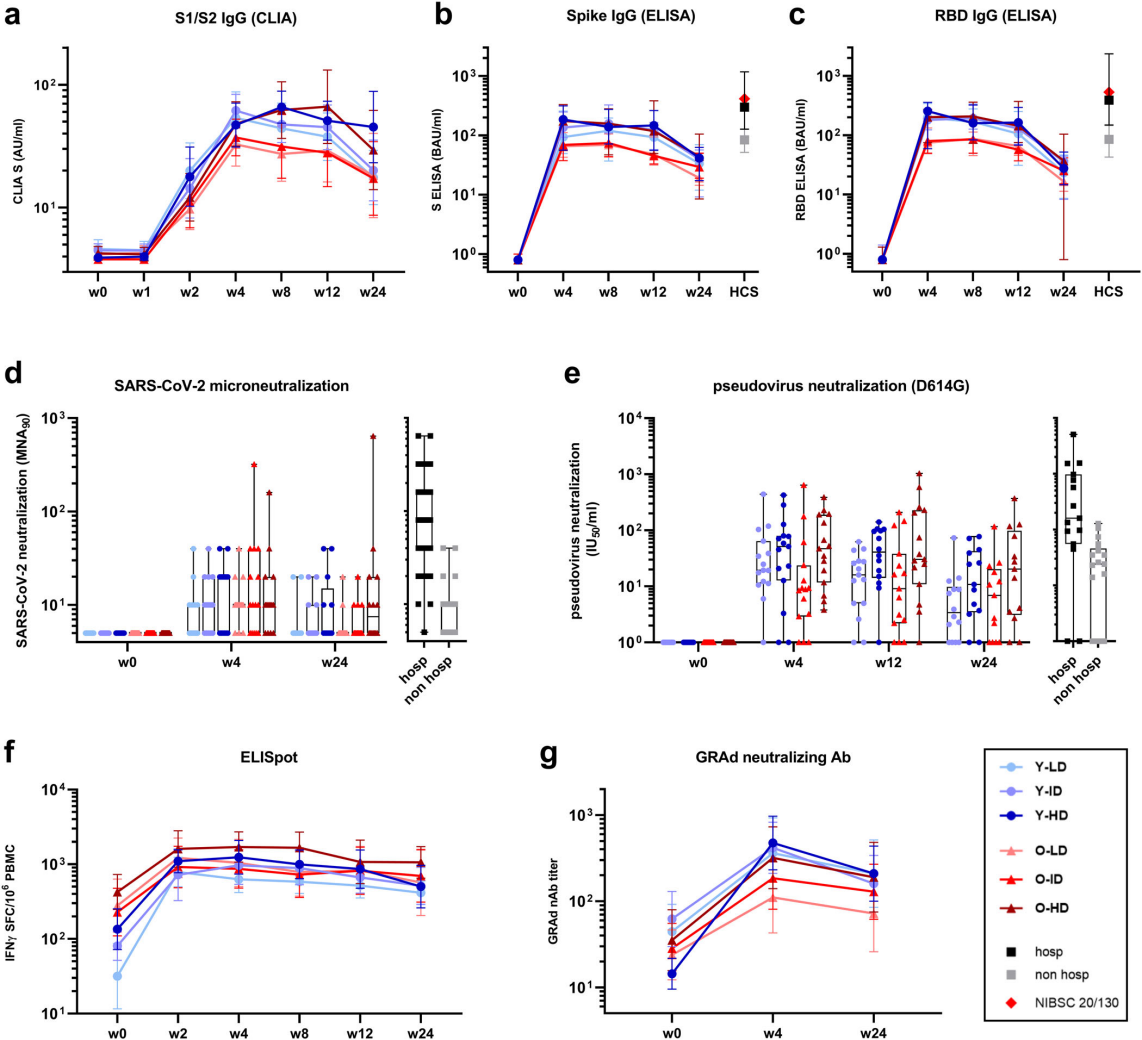
SARS-CoV-2 humoral, cellular and anti-vector response elicited by GRAd-COV2 vaccination over time. SARS-CoV-2 humoral, cellular and anti-vector response induced by GRAd-COV2 vaccination and measured at the day of vaccination (w0) and 1, 2, 4, 8, 12 and 24 weeks (w1, w2, w4, w8, w12, w24 respectively) after vaccination over 6 months follow up. *N* = 15 for each study arm. **a** IgG binding to S1-S2 measured by CLIA. Data are expressed as arbitrary unit (AU)/ml. **b** SARS-CoV-2 specific IgG measured by ELISA on recombinant full-length spike protein. **c** SARS-CoV-2 specific IgG measured by ELISA on RBD. Data in both **b** and **c** are expressed as binding antibody units (BAU)/ml. **d** Neutralizing activity detected by SARS-CoV-2 microneutralization assay. **e** Neutralizing activity detected by Spike (D614G)-pseudotyped lentivirus neutralization assay. SARS-CoV-2 neutralization titers are expressed as MNA_90_, or the reciprocal of serum dilution achieving 90% neutralization, or in International Units (IU)ml, in **d** and **e** respectively. **f** Spike-specific T cell response measured by IFNγ ELISpot on frozen PBMC. Data are expressed as IFNγ spot forming cells (SFC) per 10^6^ PBMC. **g** Neutralizing titers to the GRAd vector, measured by a neutralization assay based on SEAP reporter gene activity inhibition. Throughout, blue and red color shades identify younger (Y-circles) and older age (O-triangles) cohorts, respectively, and color tone increase with vaccine dose: low dose (LD, 5 × 10^10^ vp), intermediate dose (ID, 1 × 10^11^ vp) and high dose (HD, 2 × 10^11^ vp). Square symbols indicate human convalescent serum obtained from either previously hospitalized (hosp-dark gray) or from non-hospitalized (non-hosp-light gray) COVID-19 patients. NIBSC 20/130 research reagent plasma (red diamond) is shown for reference (panels **b** to **c**). Data are shown either as the group geometric mean and 95% confidence interval (CI) (panels **a**, **b**, **c**, **f** and **g**) or as individual points and box and whiskers indicating median and interquartile range, with whiskers ranging from min to max (panels **d** and **e**). Negative samples were assigned a value of ½ the LOD.

Spike and RBD-specific antibodies were monitored before and over the course of 24 weeks after vaccination by means of different serological assays. Seroconversion to Spike, measured by a diagnostic chemiluminescent immune assay (CLIA), occurred at week 2 post-vaccination (w2, Fig. 1a and Supplementary Fig. 1), with a higher increase observed in young respect to old subjects (w2 young/older geometric mean ratio, GMR, 1.6, *p* = 0.02). To study differences in the kinetic of immune response, CLIA results starting from week 2 were included in the mixed model for statistical analysis. “Study week” and “dose by week” interaction terms, but not “age” and “age by week”, resulted statistically significant (*p* < 0.0001 and *p* = 0.0033 for the first two terms, *p* = 0.1204 and *p* = 0.1994, for the last two terms, respectively). Spike antibody titers significantly increase from w2 to w4 (w4/w2 GMR = 3.3, *p* < 0.0001); the peak was observed at w4 for low and intermediate vaccine dose, while with high dose the titers further increased between w4 and w8 (Fig. 1a). Antibody levels remained substantially stable for 3 months, to then decline at 6 months (w12/w24 GMR = 1.9, *p* < 0.0001). Overall, there was no statistically significant difference across doses (*p* = 0.1332), while the “dose by week” interaction revealed that the high GRAd-COV2 dose induced more persistent antibody titers. Indeed, starting from w8, the high dose induced a higher anti-Spike antibody response respect to the low dose (w8: H/L dose GMR = 1.8, *p* = 0.0252; w12: H/L dose GMR = 1.7, *p* = 0.0019; w24: H/L dose GMR = 2.2, *p* = 0.0019), and was also higher than that induced by intermediate dose at w24 (H/I dose GMR = 2.2, *p* = 0.0025).

In house ELISA test for Spike and RBD specific binding antibodies, calibrated against the National Institute for Biological Standards and Control (NIBSC) 20/136, the first WHO international standard for anti-SARS-CoV-2 immunoglobulin, allowed to express the IgG concentration in binding antibody unit (BAU/ml) to facilitate comparability across studies. At peak (between w4 and w8), the geometric mean (GM) antibody level detected was between 70.4 and 155.8 BAU/ml for Spike (Fig. 1b and Supplementary Fig. 2), and between 73.4 and 187.3 BAU/ml for RBD (Fig. 1c and Supplementary Fig. 3). To corroborate the in house BAU/ml ELISA dataset, we tested sera from intermediate and high dose arms of both age cohorts using a commercially available ELISA; this assay provided slightly higher w4 peak of anti-Spike IgG (between 126.1 to 202.9 BAU/ml, Supplementary Fig. 4) but clearly in the same order of magnitude.

Statistical analysis of Spike and RBD ELISA yielded similar results than CLIA. “Study week” and “dose by week” interaction terms were statistically significant (*p* < 0.0001 and *p* = 0.0051 respectively for Spike, *p* < 0.0001, *p* = 0.0177 respectively for RBD). Spike and RBD antibody levels peaked between w4 and w8, then started declining slightly at w12 and more noticeably up to w24 (w8/w12 GMR = 1.2, *p* = 0.0001; w12/w24 GMR = 3.4, *p* < 0.0001 for Spike; w8/w12 GMR = 1.2, *p* < 0.0001; w12/w24 GMR = 4.6, *p* < 0.0001 for RBD). The high dose separated from the low dose at w4 and w12 (w4: H/L dose GMR = 1.9, *p* = 0.0374; w12: H/L dose GMR = 2.0, *p* = 0.0304 for Spike; w4: H/L dose GMR = 2.1, *p* = 0.0258; w12: H/L dose GMR = 2.2, *p* = 0.0172 for RBD), but there were no significant differences amongst doses at w24. A statistically significant interaction between “age and week” (*p* = 0.0252) was seen for RBD only, caused by a slightly different antibody kinetics between the two age cohorts, with IgG levels in younger volunteers peaking already at w4, more shifted towards w8 in older volunteers.

Although the terms “age” and “age by week” were not significant, a lower Spike and RBD antibody level in older volunteers receiving low and intermediate GRAd-COV2 dose was evident at certain study weeks with all serological assays (Fig. 1a–c, Supplementary Fig. 4).

### SARS-CoV-2 neutralizing antibody response

Measured by a microneutralization assay with live SARS-CoV-2 (Wuhan) virus, GRAd-COV2 vaccination induces a dose-dependent but highly variable level of neutralizing antibodies (MNA_90_), that contracts 6 month after vaccination (Fig. 1d). No significant differences were observed for age or dose at both study visits. However, while low and intermediate vaccine dose are associated to highly significant contraction at 6 months (Student t test for paired data w4/w24 L *p* = 0.0013, I *p* = 0.0008), it is not the case for high dose (*p* = 0.0647), suggesting better persistence of neutralization activity with higher vaccine dose.

A neutralization assay based on lentivirus pseudotyped with the ancestral (D614G) Spike glycoprotein was performed with younger and older subjects receiving GRAd-COV2 at intermediate and high dose, allowing determination of ID_50_ and conversion to IU/ml thanks to the inclusion of 20/136 reference curves (Fig. 1e). Neutralizing activity was detectable in most of younger and older age volunteers, with a statistically significant effect of vaccine dose (*p* = 0.0107) and study week (*p* < 0.0001). Specifically, the high dose induced higher neutralizing activity than intermediate dose (H/I dose GMR = 2.8). No interaction terms were statistically significant. Neutralizing antibody levels peaked at w4, remained substantially stable up to w12 and then declined significantly at 6 months (w12/w24 GMR = 2.5, *p* < 0.0001). Although the “dose by age” interaction was not statistically significant (*p* = 0.1192), a higher vaccine dose was beneficial for better neutralizing activity in older age volunteers (H/I dose GMR 5.1, *p* = 0.0041) while less so in younger (H/I dose GMR 1.5, *p* = 0.4439). GM neutralizing Ab levels at w4 ranged between 9.8 IU/ml (older age, intermediate dose) to 42 IU/ml (older age, high dose).

Overall, binding and neutralizing Ab titers in GRAd-COV2 vaccinees are similar to those measured in COVID-19 non-hospitalized patients within 2 months from symptoms onset (Fig. 1b–e and Supplementary Fig. 5).

### T-cell response

T cells cross-recognizing SARS-CoV-2 Spike peptides, most probably representing memory responses to seasonal coronaviruses, are already detectable at baseline in many subjects (Fig. 1f and Supplementary Fig. 6) and interestingly at higher levels in older subjects *(p* < 0.0001 by ANOVA). GRAd-COV2 vaccination induced potent and persistent IFNγ-secreting T cell responses to Spike, peaking already 2 weeks after vaccination and sustained for the whole 6 months follow-up (Fig. 1f). Statistical analysis by linear mixed model with the baseline level as the covariate showed a statistically significant effect of the covariate and the time factors (both *p*-values < 0.0001). T cell responses were stable between 2 and 4 weeks and started to decrease from w8 with a mild but continuous slope (w4/w8 GMR = 1.2, *p* = 0.0140; w8/w12 GMR = 1.2, *p* = 0.0045; w12/w24 GMR = 1.2, *p* = 0.0002). Despite a decline in T cell response over time, circulating Spike-specific T cells at 6 months post-GRAd-COV2 vaccination were still higher than at baseline in all groups (Student t-test for paired data comparing w24 to baseline showed *p* < 0.01 in all dose/age groups). Additionally, Spike T cell response of similar magnitude and kinetics were induced in both age cohorts, with no vaccine dose-dependence. The Spike T cell fold increase post vaccination was generally lower in subjects with higher pre-existing T cell responses, suggesting limited expansion capacity.

### Antivector-specific neutralizing antibodies

The favorable low prevalence of anti-GRAd vector (cross)-neutralizing antibodies at baseline was previously reported and discussed^48^, with only 11% of subjects with neutralization titers >200 at study entry. GRAd-COV2 vaccination induced or boosted such neutralizing response in most volunteers (Fig. 1g and Supplementary Fig. 7). The ANCOVA model including levels at w0 as covariate showed a statistically significant effect for age and vaccine dose (*p* = 0.0199 and *p* = 0.0133, respectively) in addition to the covariate (*p* < 0.0001): the highest vaccine dose most prominently induced anti-vector neutralization (H/L dose GMR = 2.5, *p* = 0.0081; H/I dose GMR = 2.1, *p* = 0.0317). A more vigorous induction/expansion of such responses was seen in younger age volunteers compared to older age cohort (Y/O GMR = 2.0).

Among 1- and 6-months post immunization the GM anti-vector responses were significantly contracted between 1.5-fold to 2.6-fold in the different study arms irrespective of age or vaccine dose (all w4/w24 by Student t test for paired data *p* = <0.0003), but remaining higher than at baseline.

### SARS-CoV-2 infection and exposure of vaccinated subjects

There was a high circulation of SARS-CoV-2 in Italy throughout the study follow up (September 2020 to May 2021), and one volunteer was infected with SARS-CoV-2. The subject (102044) was in the GRAd-COV2 high dose, younger age cohort and developed mild symptomatic illness. Additional 22 volunteers (Supplementary Table 3) reported SARS-CoV-2 exposure, amongst which 4 from a cohabitant; all were asymptomatic and had negative at SARS-CoV-2 molecular testing on nasopharyngeal swabs.

**Fig. 2:**
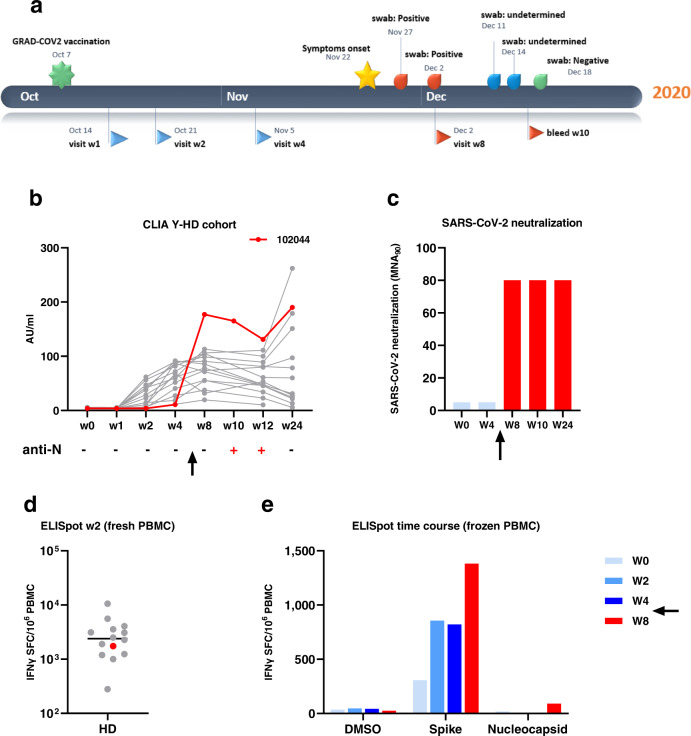
Immune response before and after SARS-CoV-2 infection in a GRAd-COV2 vaccinated volunteer. **a** Timeline detailing the SARS-CoV-2 infection case in terms of vaccination, in-study and out-of-study visits, symptoms onset and swabs outcome until resolution. **b** IgG binding to S1-S2 measured by CLIA in arm 3 (high dose vaccine in younger age volunteers), with IgG kinetics in the COVID-19 case depicted in red and all remaining volunteers in gray for context. Below each study visit indicated on x axis, the outcome of a qualitative CMIA detecting anti-nucleocapsid (N) IgG is reported for the COVID-19 case. **c** SARS-CoV-2 neutralizing activity time course in serum from the COVID-19 case, detected by MNA. **d** T cell response to Spike in arm 3 detected by IFNγ ELISpot on freshly isolated PBMC 2 weeks after vaccination, with the COVID-19 case depicted in red and all remaining volunteers in gray for context. The horizontal line is set at median. **e** Spike and N T-cell response time course in the COVID-19 case, detected by IFNγ ELISpot on frozen PBMC. DMSO, the peptide pools diluent, represents the assay negative control. The arrow in panels **b**, **c** and **e** highlight timing of SARS-CoV-2 infection. Postinfection visits data in panels **c** and **e** are depicted in red.

The infected subject reported symptoms of fever, chills, headache and loss of taste and smell at 6 weeks postvaccination. The subject then subsequently tested negative 1 month later (Fig. 2a). The postvaccination Spike IgG antibody and neutralization titers were poor in this subject, with barely detectable seroconversion and no SARS-CoV-2 neutralizing activity detectable at the week 4 visit preceding infection (Fig. 2b, c). The subject however had a good Spike T cell response at w4 postvaccination (Fig. 2d). Spike binding and SARS-CoV-2 neutralizing Ab were potently boosted upon infection (Fig. 2b, c), along with T cell response (Fig. 2e), suggesting that even if sub-optimally, GRAd-COV2 vaccination had primed both humoral and cellular response in this subject. Seroconversion to SARS-CoV-2 Nucleocapsid occurred 1 month after infection (Fig. 2b), but was back to negative 3 months later at the 6 months study visit, when Spike binding and neutralizing Ab were still high.

## Discussion

The second year of SARS-CoV-2 pandemic is reaching an end, but the global situation is far from being finally under control although billions of people got vaccinated in the world. Moreover, the low rate of vaccination in some regions (e.g., Africa) could lead to the continued emergence of novel VOC requiring additional booster doses to face the decline of vaccine-mediated protection over time. In this scenario, the development and clinical validation of additional efficacious vaccines is still essential. Here we report on the complete clinical and immunological follow-up from the phase 1 trial of GRAd-COV2, a COVID-19 vaccine candidate based on a gorilla adenovirus. A single administration of GRAd-COV2 at all tested doses was well tolerated, and induced Spike/RBD binding and neutralizing antibodies sustained for at least 3 months. Immune response kinetics in older age volunteers were similar as in younger population. A non-significant trend for higher antibody titers was observed in younger volunteers, with the exception of the older cohort arm receiving the highest vaccine dose (2 × 10^11^ vp) whose responses were superimposable to those in younger.

SARS-CoV-2 antibodies, particularly the neutralizing component, decline at 6 months after GRAd-COV2 vaccination, as now well described for other COVID-19 approved vaccines;^22,26,28–30,56^ this points to the need of booster doses for GRAd-COV2 as for all other vaccines, to maintain high antibody titers and to ensure enough activity against circulating VOC. In the ongoing GRAd-COV2 phase 2 clinical trial it will be important to assess if and to what extent the magnitude and kinetics of GRAd-COV2-induced antibodies can be improved by administration of a second GRAd-COV2 dose.

Initial reports aimed at establishing immunological correlates of protection (CoP) for approved COVID-19 vaccines have been published^57–59^, taking advantage of the recent introduction of the first WHO international standard for SARS-CoV-2 IgG that enabled expressing serological measures coming from different assays in common units of measurement. These studies have major limitations, mainly consisting in limited median follow up (90–130 days postsecond vaccine dose^57,58^) and in the fact that they were run in a context of ancestral (Wuhan/Alpha variant) circulating virus. Therefore, the proposed CoP may predict at best short-term protective efficacy against symptomatic Wuhan infection. However, the outcome of these studies can be precious to evaluate the potential for protective efficacy of novel vaccines still in development. Specifically, in a global context where classical placebo-controlled phase 3 efficacy studies are deemed extremely difficult if not impossible to implement for practical and ethical considerations, such correlates enable extrapolation of efficacy from immunogenicity data for second-wave vaccine candidates that cannot achieve clinical efficacy evaluation.

Similar findings emerged from these initial studies: both Spike/RBD binding and neutralizing antibody levels were established as valid correlates of risk, and no firm absolute “protective threshold” could be established so far; rather, a continuum model is suggested where the probability of breakthrough SARS-CoV-2 infection decrease with increasing levels of the vaccine-induced immune response, but with substantial individual variation. In addition, these studies suggested that the median level of immune response that a novel vaccine should induce at peak after primary vaccination cycle associated to predicted efficacy between 60 and 90% against symptomatic infection are rather low, in the order of few hundreds BAU/ml for binding, and in the range of 4 to 140 IU/ml for neutralizing antibodies by pseudovirus neutralization.

Here we show that a single administration of GRAd-COV2 at peak (w4-8) after vaccination induces anti-Spike and anti-RBD IgG concentrations (geometric mean) in the range of 70-180 BAU/ml with in house developed ELISAs, reaching 200 BAU/ml with a commercial ELISA kit; the geometric mean of neutralizing antibodies by means of pseudoneutralization assay were between 10 and 40 IU/ml, depending on vaccine dose. These peak immune response levels are comparable to those induced by approved COVID-19 vaccines based on adenoviral vector platform (ChAdOx1 nCoV-19/AZ1222 and Ad26.COV2.S^59^), and would predict an efficacy around 70–80% according to the two reports mentioned^57,58^. Additionally, immune response levels are expected to further improve with a two-dose GRAd-COV2 regimen.

We also confirm here that GRAd-COV2 vaccination induces a potent T cell response that is sustained to high levels in the months following vaccination, and this is regarded as an important feature for vaccine-mediated protection from COVID-19 severe disease. A similar kinetics of potent and durable T cell response to Spike upon a single vaccine administration with minimal contraction at 6–8 months follow up was also reported for ChAdOx1 nCoV-19/AZ1222^60^ and for Ad26.COV2.S^21^. Moreover, it has been shown by us and by others that CD4 and CD8 response either induced by vaccination or SARS-CoV-2 infection is highly cross-reactive and able to recognize Spike from all circulating VOC, including Omicron^48–54^. Pre-existing Spike T cell response expanded more vigorously in younger compared to older age volunteers upon vaccination, consistently with a reduced proliferation capacity of potentially senescent memory T cells^61^.

The proficiency in inducing remarkable T cell responses to the encoded antigen already after a single administration is a distinctive feature of adenoviral vaccines, which in light of the VOC cross-reactivity makes them still a very desirable option either as stand-alone primary vaccination cycle or in heterologous prime-boost combinations with vaccines based on different platform technologies (mRNA or protein). Adenoviral vectors, along with robust immune responses to the encoded transgene, also induces neutralizing antibodies to its own viral components, as shown here for GRAd-COV2. Such anti-vector immunity may hamper vaccine immunogenicity when there is a need for repeated administration over a short interval of time to counteract waning humoral immunity to the vaccine antigen, as in the case of SARS-CoV-2. The possibility to mix and match vaccines from different platform technologies and/or alternate vaccines based on different adenovirus serotypes may represent the best way to exploit and synergize specific beneficial features associated to each vaccine platform, as suggested by a large body of preclinical and clinical experience with heterologous prime boost strategies in challenging vaccine research fields such as HIV, Tuberculosis, malaria or influenza^62,63^. Recent literature on clinical trials or real-life studies of such heterologous prime-boost regimens has convincingly shown their notable immunogenicity and efficacy^39–46,64,65^, and suggests that using first an Ad-based vaccine optimally induces T cell response and primes antibody and B cells that can be greatly amplified by booster vaccinations, potentially offering adequate (cross)-protection in the time frame between first and second vaccination or between primary cycle and booster dose. This is also illustrated well by the single case of SARS-CoV-2 infection in our phase 1 trial, which was conducted during a time period of very high SARS-CoV-2 circulation in Italy. In this subject, the GRAd-COV2 vaccine induced suboptimal antibodies but a good T cell response, the latter of which may have resulted in mild symptoms. Both Spike-specific antibodies and T cells were rapidly and robustly boosted by the SARS-CoV-2 infection.

The compatibility of GRAd-COV2 candidate vaccine with currently approved COVID-19 vaccines has been verified serendipitously in the few participants from the present RT-CoV-2 study who opted for receiving COVID-19 vaccination before the last planned study visit at w24; indeed, we recently reported^66^ that AZ1222 or BNT-162b2 vaccination more than 3 months after a single GRAd-COV2 administration potently boosted both humoral and cellular response to Spike, suggesting that a novel, potent adenoviral vectored vaccine could be successfully combined with alternative vaccines based on either different adenoviral serotype(s) or on mRNA platform.

Only the implementation in mass-vaccination campaign of the two COVID-19 vaccines ChAdOx1 nCoV-19 and Ad26.COV2.S could led to the recognition of the very rare VITT syndrome^37,38^, that has casted a serious shadow on the adenoviral vector platform as a whole. The mechanism underlying this syndrome has not yet been elucidated, and many hypotheses have been advanced^67^ related to impurities in the vaccines suspension^68,69^ or aberrant transcription of the Spike protein from encoded DNA^70^. A direct link of VITT to the adenovirus particles per se has not been conclusively established; however recent studies demonstrating an electrostatic interaction between Ad capsids and PF4, the target of the pathogenic autoantibodies that are present at high levels in the VITT patients^71,72^, point towards that direction. It is intriguing that no VITT events have been reported for other worldwide employed COVID-19 vaccines based on adenovirus such as Convidecia (human Ad5) or Sputnik (hAd5 and Ad26), but this may be related to poor/non-existing pharmacovigilance in the countries adopting these vaccines. GRAd vector, like Ad5, is classified as a group C adenovirus^55^, differently from ChAdOx1 (group E) and Ad26 (group D). Therefore, understanding if and to what frequency VITT occurs upon Ad5 vaccination would help understanding if it should be considered a class-effect of the adenoviral vector spectrum or not. It will be even more critical to clarify all aspects of this syndrome, in order to adopt clinical countermeasures or engineer the next generation vectors to eventually rescue an otherwise highly immunogenically potent, manageable and versatile vaccine platform.

The immunogenicity dataset shown here for GRAd-COV2 upon a single administration is definitely encouraging, and will be soon corroborated by the ongoing phase 2 study (NCT04791423) safety and immunogenicity results in a much larger sample size. Additional desirable properties of adenoviral vector-based vaccines such as the potential for storage at refrigerator temperature, a cheaper price per dose and a manufacturing process that is easy to scale up and reasonably amenable for tech-transfer to good manufacturing practice (GMP) facilities in LMIC, make GRAd-COV2 an attractive candidate for addition in the current portfolio of COVID-19 vaccines.

## Methods

### Study design

GRAd-COV2 is a gorilla-derived adenovirus vector encoding the full length, pre-fusion stabilized Spike protein (Wuhan strain)^55^. GRAd-COV2 was manufactured by ReiThera under GMP conditions in the proprietary cell line ReiCell35S (a suspension-adapted packaging cell line derivative of HEK293) and purified by an extensive downstream process including host cell DNA precipitation, depth filtration, two chromatographic purification steps followed by nuclease digestion and ultrafiltration.

This study is a phase 1, dose-escalation, open-label clinical trial designed to determine the safety and immunogenicity of GRAd-COV2. The study included two age cohorts, of either younger (18 to 55 years) or older (65 to 85 years) adults. No formal sample size calculation was carried out. Each cohort consisted of 3 arms of 15 volunteers each, for assessing a single administration at three different doses of GRAd-COV2: low dose (5 × 10^10^ vp), intermediate dose (1 × 10^11^ vp), and high dose (2 × 10^11^ vp). Eligible participants were subjects who meet age criteria of either cohort with no history of COVID-19, no laboratory findings suggestive of current or previous infection with SARS-COV-2 and who have attended the screening visit no more than 21 days before vaccination. Volunteers were expected to attend several visits: day 2, week 1, week 2 and week 4, week 8, week 12 and week 24 after vaccination. During the visit, the volunteers underwent blood sampling and medical evaluation. Participants also discussed with a doctor any potential AEs. The study was supervised by an independent data safety monitoring board. Volunteers recorded local and systemic reactions on a diary card for 28 days. The AEs severity and relatedness with vaccination were assessed by the medical team in each center. Detailed study description and study protocol can be found in Lanini et al.^48^. Of note, 8 subjects adhered to National COVID-19 vaccination campaign, to receive approved BNT162b2 or ChAdOx1 nCoV-19 vaccines between w12 and w24 visits (outlined in Agrati et al.^66^). Their Ab and T cell data at w24 were removed from current report (main figures and statistical analysis), which therefore only reflects GRAd-COV2 immunogenicity. They are anyway shown in Supplementary Figs. 1 to 3, where immunogenicity data are shown for individual volunteers.

As comparator for immunogenicity analysis, we used two independent sets of anonymized serum specimens from COVID-19 patients either hospitalized or recovering from mild symptomatic disease, collected 20 to 60 days after symptom onset. A reference research reagent, anti-SARS-CoV-2 plasma (NIBSC, code 20/130) acquired from a donor who recovered from COVID-19, was included in most serological assays as a positive control. The first WHO International Standard for anti-SARS-CoV-2 immunoglobulin (human) NIBSC code: 20/136, pooled plasma obtained from eleven individuals recovered from SARS-CoV-2 infection, was used to calibrate ELISA and pseudoneutralization assay. Both reagents were obtained from the National Institute for Biological Standards and Control, UK.

### Ethical statement

All participants provided written informed consent before enrolment. The trial was conducted at the National Institute for Infectious Diseases Lazzaro Spallanzani (INMI) in Rome and at Centro Ricerche Cliniche in Verona (CRC-Verona). The study was conducted according to the Declaration of Helsinki, and approved by the Italian Regulatory Drug Agency (AIFA) and the Italian National Ethical Committee for COVID-19 clinical studies (ClinicalTrials.gov NCT04528641; EudraCT 2020-002835-31). Serum samples from convalescent patients who resolved SARS-CoV-2 infections came from residual specimens used for diagnostic purposes and were utilized according to INMI protocols for observational studies approved from internal ethical committee.

### SARS-CoV-2 anti-Spike protein and anti-Nucleocapsid protein IgG high throughput Chemiluminescence Immunoassay

DiaSorin LIAISON SARS-CoV-2 S1/S2 IgG CLIA detecting anti-S1/S2 IgG on LIAISON XL analyzers, and Abbott SARS-CoV-2 assay chemiluminescence microparticle assay (CMIA) detecting anti-Nucleocapsid (N) protein IgG run on Abbott ARCHITECT i2000sr, were performed according to manufacturer’s instructions. For Spike, IgG antibody concentrations are expressed as arbitrary units (AU) per ml, with AU/ml >15 considered positive. For N (qualitative), an index value sample cut-off >1.4 is considered positive.

### Spike and RBD ELISA

SARS-CoV-2 Spike and RBD ELISAs were performed essentially as previously reported^48^ with modifications to express data in BAU/ml. Coated proteins were either full-length soluble prefusion stabilized Spike protein expressed in Expi293 cells (ReiThera, 500 ng/well) or a recombinant RBD expressed in HEK293 cells (ACROBiosystems, 250 ng/well). Proteins were diluted in PBS and coated in 100 μl volume on NUNC Maxisorp plates (Thermo Fisher Scientific) overnight at 4 °C. The following day, plates were washed with PBS 0.05% Tween-20 (PBS-T), then blocked with PBS-T + 3% non-fat dry milk for 1.5 h at 25 °C with shaking. A reference curve of the WHO international standard (NIBSC code 20/136, 1000 BAU/ml) prepared in PBS-T + 1% nonfat dried milk and made of 8 × 2.5-fold serial dilutions starting at 1:100 (from 10 to 0.016 BAU/ml) was added to each plate. Sera from GRAd-COV2 vaccinated volunteers or from COVID-19 patients were tested at a fixed dilution in PBS-T + 1% non-fat dried milk of 1:500, defined during assay optimization, while baseline sera were tested at 1:100 dilution for increased sensitivity. Diluted sera were plated in 100 μl volume and plates were incubated for 2 h at 25 °C with shaking, then washed and incubated with 1:2000 diluted anti-human IgG (Fc-specific, Sigma-Aldrich) for 1 h at 25 °C with shaking. Plates were then washed and incubated with alkaline phosphatase substrate SigmaFast (Sigma-Aldrich) at 25 °C. Absorbance was read at 405 and 620 nm using EnSight multiple plate reader (Perkin Elmer). The assay LOQ was set at 1.6 BAU/ml (i.e. last standard curve point 0.016 * 100, or the highest serum dilution tested). Anti-SARS-CoV-2 research reagent plasma (NIBSC code 20/130, diluted 1:500) was used as positive control in each experiment. Concentrations in BAU/ml were then obtained by interpolating from the NIBSC 20/136 standard curve the resulting optical densities (OD) data, multiplied by serum dilution factor.

Kantaro Quantitative SARS-CoV-2 IgG Antibody RUO Kit (Biotechne, MN, US) was performed following manufacturer’s instruction. Inactivated sera (56 °C for 1 h), were tested at a final dilution of 1:200. LLOQ of the Spike quantitative assay was 3.2 AU/ml for the lower limit and ULOQ 160 AU/ml. BAU/ml were calculated by multiplying AU/ml values by the conversion factor of 0.0235 and by the final dilution (i.e. 200), according to manufacturer’s indication.

### SARS-CoV-2 microneutralization assay (MNA)

Serum samples collected from vaccinated volunteers or convalescent COVID-19 patients were heat-inactivated at 56 °C for 30 min, and titrated in duplicate in 7 two-fold serial dilutions (starting dilution 1:10). Equal volumes of 100 TCID50 (Tissue Culture Infectious Dose 50%) of SARS-CoV-2 (strain 2019-nCoV/Italy-INMI1; GISAID accession ID: EPI_ISL_412974) and serum dilutions were mixed and incubated at 37 °C 5% CO2 for 30 min. Subsequently, 96-well tissue culture plates with sub-confluent Vero E6 cell (ATCC) monolayers were infected with 100 μl/well of virus-serum mixtures and incubated at 37 °C and 5% CO2. To standardize inter-assay procedures, positive control samples showing high (1:160) and low (1:40) neutralizing activity were included in each MNA assay. After 48 h, microplates were observed by light microscope for the presence of cytopathic effect (CPE). The supernatant of each plate was carefully discarded and 120 μl of a Crystal Violet solution containing 2% formaldehyde was added to each well. After 30 min, the fixing solution was removed by washing with tap water and cell viability measured by photometer at 595 nm (Synergy HTX Multi-Mode Microplate Reader, Biotek). The highest serum dilution inhibiting at least 90% of the CPE was indicated as the neutralization titer and expressed as the reciprocal of serum dilution (MNA_90_).

### Pseudovirus neutralization assay

Samples from vaccinated volunteers were analyzed for neutralizing antibodies against SARS-CoV-2 with a pseudovirus neutralization assay using a luciferase reporter–based pseudotyped lentiviral virus system coated with SARS-CoV-2 D614G Spike protein. The construction of the plasmids bearing the Spike cassette deleted of 19 amino acids at the cytoplasmic tail used for the pseudotyping, the generation and the titration of the pseudovirus preparations were described previously^73^. For the pseudoneutralization assay, Vero E6 cells were maintained, plated and infected in DMEM + 10% FBS + 2 mM Glutammine + 1% P/S. Cells were seeded at a density of 1.5–2 × 10^4^ cells/well in a 96-well Isoplate (PerkinElmer) 24 h before the neutralization.

Three-fold serial dilutions of tested sera and WHO international standard 20/136 (used as positive control on each plate), all heat inactivated at 56 °C for 45 min, were incubated with equal volumes of pseudovirus preparation (diluted to generate 4.5–7.0 × 10^5^ RLU per well) for 30 min at 37 °C 5% CO_2_ in a U-bottom 96 well (Costar), in triplicate. Serum/virus mixtures were then transferred on the Vero E6 cell monolayers. After 48 h, cells were lysed and subjected to luciferase assay by using the Britelite plus Report Gene Assay System (PerkinElmer); resulting relative luminescence units (RLU) were measured at EnSight plate reader (Perkin Elmer) and used to determine percentages of neutralization, calculated for each plate as the percentage of signal reduction relative to the virus-only control. Dose-response curves were generated with a nonlinear regression model, and titers reported as the serum dilution required to achieve 50% neutralization (IC_50_), as described^74^. Tested sera were analyzed with an input dilution of 1:80. Those that resulted weakly positive or with very low extrapolated titer were tested again with an input dilution of 1:20. Thus, 20 is the lower limit of quantification and samples that do not neutralize at the 50% level are expressed as <20.

To convert the IC_50_ of samples to International Units, the sample’s potency is calculated as a ratio relative to that determined for the international standard NIBSC 20/136, which has been assigned an arbitrary unitage of 1000 IU / ml. Unitage for a tested sample was therefore expressed as:

$${{{\mathrm{Sample}}}}\;{{{\mathrm{units}}}} = 1000\;{{{\mathrm{IU}}}}/{{{\mathrm{ml}}}} \ast \left( {{{{\mathrm{sample}}}}\;{{{\mathrm{IC}}}}_{50}/20/136\;{{{\mathrm{IC}}}}_{50}} \right)$$

The calibrant IC_50_ titer was determined by averaging the titers of 20/136 reference present on each plate of all experiments with which the final dataset was generated.

### GRAd neutralizing antibody assay

Briefly, 8 × 10^4^ HEK293 cells per well were seeded in 96 well plates the day before the assay. GRAd vector encoding the reporter gene secreted alkaline phosphatase (SEAP) at a pre-optimized multiplicity of infection (MOI) was preincubated for 1 h at 37 °C alone or with serial dilutions of control or test serum samples. Samples were then added to the 80–90% confluent HEK293 cells. After incubation for 1 h at 37 °C, the serum/infection mix was removed and replaced with 10% fetal bovine serum (FBS, Cytiva HyClone) in Dulbecco’s Modified Eagle Medium (DMEM, Thermo Fisher). SEAP expression was measured 24 h later in cell supernatant by means of the chemiluminescent substrate from the Phospha-Light kit (Applied Biosystems). Neutralization titers were defined as the dilution at which a 50% reduction of SEAP activity from serum sample was observed relative to SEAP activity from virus alone.

### Peripheral blood mononuclear cells (PBMC) isolation, freezing and thawing

Peripheral venous blood (40 ml) collected in lithium-heparin Vacutest tubes (Kima) were isolated by standard density gradient centrifugation (Histopaque 1077, Sigma-Aldrich). After separation, PBMCs were suspended in RPMI-1640 (Sigma-Aldrich) supplemented with 10% heat-inactivated highly defined FBS (Cytiva HyClone), 2 mmol/l L-glutamine, 10 mmol/l HEPES buffer (N-2-hydroxyethylpiperazine-N-2-ethane sulfonic acid, Sigma-Aldrich), 100 U/ml penicillin, and 100 µg/ml streptomycin (Gibco), hereafter termed R10, counted using Guava Muse (Luminex) and frozen in FBS plus 10% dimethyl sulfoxide (DMSO). PBMCs were thawed quickly in 37 °C water bath with thawing medium [CTL Wash supplemented medium in RPMI-1640 (Sigma-Aldrich) and supplementing with L-glutamine (Gibco) and Benzonase (Merck) 50U/ml]. After one wash, PBMC were resuspended into 50 ml polypropylene vented cap tubes with prewarm R10 medium, counted and rested at 2 × 10^6^ cells/ml at 37 °C and 5% of CO_2_ for at least 16 h. PBMCs were then counted, resuspended at 4 × 10^6^ cell/ml in R10 medium and used in ELISpot assays.

### IFNγ ELISpot assay

A set of 316 15mer peptides overlapping by 11 amino acids (synthetized by Elabscience Biotech Inc, and distributed by TEMA RICERCA), designed to cover the full-length Spike protein, was arranged into 2 pools (S1 and S2). The frequency of IFNγ-producing T cells was assessed by enzyme-linked immunosorbent spot-forming cell assay (ELISpot). Thawed and rested PBMC were plated at 2 × 10^5^ cells/well in ELISpot plates (Human IFNγ ELISpot plus kit; Mabtech), and stimulated with the two Spike peptide pools for 18 to 20 h (3 μg/ml each peptide final concentration) at 37 °C with 5% CO2. At the end of incubation, the ELISpot assay was developed according to manufacturer’s instructions. Results are expressed as IFNγ spot forming cells (SFC) per 10^6^ PBMCs in Spike-stimulated cultures after subtracting background from wells stimulated with DMSO, the peptide diluent. Results were deemed valid when spontaneous IFNγ secretion (response to DMSO) was ≤ 288 SFC per million PBMC, corresponding to the mean+1 standard deviation of DMSO responses throughout the whole dataset. For statistical analysis, 10 subjects were excluded due to >50% of tested time points with invalid results.

### Statistical analysis

The original immunogenicity data were normalized by means of the natural logarithm (log) transformation.

For each immunogenicity endpoint with at least 3 timepoints after baseline, log transformed values measured at all post-baseline assessments were analyzed using a linear mixed model for repeated measures including dose, age, week of assessment and their two-way interactions as fixed effects. In case of baseline values among subjects different from all negative (this was the case for ELISpot and GRAd neutralizing antibodies), the baseline log transformed values were used as covariate.

The restricted maximum likelihood with the Newton-Rapson solution was used for the maximization. An unstructured covariance matrix was selected, based on the Akaike information criterion (AIC), which is a tool to assess the model fit (a smaller AIC value indicates a “better” model).

For each immunogenicity endpoint with less than 3 timepoints after baseline, the values observed at each of the post-baseline timepoints were analyzed (i.e., week 4 and week 24). MNA_90_ log transformed values were analyzed by means of an analysis of variance (ANOVA) model including dose, age, and their interaction as fixed effects. Anti-GRAd Neut log transformed values were analyzed by means of an analysis of covariance (ANCOVA) model including the same factors described for the ANOVA model plus the log transformed baseline levels as covariate.

Occasionally, comparisons between antibody levels at baseline versus other study weeks were carried out with the Student’s t-test for paired data.

The least square mean differences between the factors being compared and the corresponding p-values were estimated from the described models; point estimates were exponentiated to obtain geometric mean ratio (GMR) estimates. No imputation of missing values was implemented, i.e., only observed data were included in the analysis. Under the missing at random assumption, the linear mixed model for repeated measures provides an unbiased estimate of the factor effects that would have been observed if all subjects had completed the study. For each timepoint (week 4 and week 24), the ANOVA/ANCOVA models included only subjects with an observation at that time. No adjustment for multiplicity of testing was applied, i.e., all tests were considered statistically significant if the relevant p-value was less than 0.05 (two-sided).

The statistical analysis was carried out with the NCSS 2021 (v21.0.2.) software.

## Supplementary information

Supplementary Material

## Data Availability

All data are presented in the main text or the supplementary materials and are available upon reasonable request from the corresponding author.
