# Supplementary material for: Safety and immune response kinetics of GRAd-COV2 vaccine: phase 1 clinical trial results
Source: NPJ Vaccines. 2022 Sep 24;7:111. doi: 10.1038/s41541-022-00531-8 (PMC9509317; doi:10.1038/s41541-022-00531-8)
Supplement: Supplementary file 1 — Supplementary Material [file 41541_2022_531_MOESM1_ESM.pdf]

Safety and immune response kinetics of GRAd-COV2 vaccine: phase 1 clinical trial results

Chiara Agrati[1†], Concetta Castilletti[1†§], Simone Battella[2], Eleonora Cimini[1], Giulia Matusali[1], Andrea Sommella[2], Alessandra Sacchi[1], Francesca Colavita[1], Alessandra M. Contino[2], Veronica Bordoni[1], Silvia Meschi[1], Giulia Gramigna[1], Federica Barra[2], Germana Grassi[1], Licia Bordi[1], Daniele Lapa[1], Stefania Notari[1], Rita Casetti[1], Aurora Bettini[1], Massimo Francalancia[1], Federica Ciufoli[2], Alessandra Vergori[1], Serena Vita[1], Michela Gentile[2], Angelo Raggioli[2], Maria M. Plazzi[1], Antonella Bacchieri[3], Emanuele Nicastri[1], Andrea Antinori[1], Stefano Milleri[4], Simone Lanini[1], Stefano Colloca[2], Enrico Girardi[1], Roberto Camerini[2], Giuseppe Ippolito[1#], Francesco Vaia[1], Antonella Folgori[2], Stefania Capone[2*]

Supplementary material

Supplementary table 1: Overview of adverse events

Adverse Events Categories<br>Number (%) of Subjects	Arm 1 LD (N=15)<br>n (%) #E	Arm 2 ID (N=15)<br>n (%) #E	Arm 3 HD (N=15)<br>n (%) #E	Younger age Total (N=45)<br>n (%) #E	Arm 4 LD (N=16)<br>n (%) #E	Arm 5 ID (N=15)<br>n (%) #E	Arm 6 HD (N=15)<br>n (%) #E	Older age Total (N=46)<br>n (%) #E	ALL (N=91)<br>n (%) #E
Any TEAE	12 (80.0)<br>60	14 (93.3)<br>76	14 (93.3)<br>71	40 (88.9)<br>207	13 (81.3)<br>59	9 (60.0)<br>39	9 (60.0)<br>39	31 (67.4)<br>137	71 (78.0)<br>344
Any TEAE related to study drug	12 (80.0)<br>48	13 (86.7)<br>53	13 (86.7)<br>63	38 (84.4)<br>164	12 (75.0)<br>44	9 (60.0)<br>24	8 (53.3)<br>36	29 (63.0)<br>104	67 (73.6)<br>268
Any treatment-emergent SAE	0	0	0	0	0	0	0	0	0
Any TEAE leading to study discontinuation	0	0	0	0	0	0	0	0	0
Any AESI	0	0	0	0	0	1 (6.7)<br>1	0	1 (2.2)<br>1	1 (1.1)<br>1
Any solicited adverse reactions emerged in the first 7 days post dose	12 (80.0)<br>42	13 (86.7)<br>56	13 (86.7)<br>59	38 (84.4)<br>157	11 (68.8)<br>40	9 (60.0)<br>24	8 (53.3)<br>38	28 (60.9)<br>102	66 (72.5)<br>259
TEAE leading to death	0	0	0	0	0	0	0	0	0

AESI=adverse events of special interest, N=number of subjects in the SAF, n=number of subjects with at least one AE within category. All subjects are counted only once per AE category, SAE=serious adverse event, SAF=Safety Analysis Population, TEAE=treatment-emergent adverse event, #E=number of all events within category, %=n/N, LD= Low dose ($5x10^{10}$ viral particles), ID= Intermediate Dose ($1x10^{11}$ viral particles), HD= High Dose ($2x10^{11}$ viral particles).

Supplementary table 2: Summary of Treatment-emergent Solicited Adverse Reactions Emerged in 7 days Post dose by Preferred Term (SAF)

Adverse Events Categories	Arm 1 LD (N=15)	Arm 2 ID (N=15)	Arm 3 HD (N=15)	Younger age Total (N=45)	Arm 4 LD (N=16)	Arm 5 ID (N=15)	Arm 6 HD (N=15)	Older age Total (N=46)	ALL (N=91)
**Number (%) of Subjects**	**n (%) #E**	**n (%) #E**	**n (%) #E**	**n (%) #E**	**n (%) #E**	**n (%) #E**	**n (%) #E**	**n (%) #E**	**n (%) #E**
Injection site pain	10 (66.7) 10	12 (80.0) 13	10 (66.7) 10	32 (71.1) 33	2 (12.5) 2	1 (6.7) 1	4 (26.7) 4	7 (15.2) 7	39 (42.9) 40
Fatigue	8 (53.3) 10	7 (46.7) 8	10 (66.7) 11	25 (55.6) 29	7 (43.8) 9	2 (13.3) 2	4 (26.7) 4	13 (28.3) 15	38 (41.8) 44
Headache	5 (33.3) 6	6 (40.0) 9	10 (66.7) 10	21 (46.7) 25	6 (37.5) 8	3 (20.0) 3	2 (13.3) 2	11 (23.9) 13	32 (35.2) 38
Pyrexia	3 (20.0) 3	4 (26.7) 5	11 (73.3) 11	18 (40.0) 19	4 (25.0) 4	4 (26.7) 4	4 (26.7) 4	12 (26.1) 12	30 (33.0) 31
Chills	1 (6.7) 1	1 (6.7) 1	6 (40.0) 6	8 (17.8) 8	4 (25.0) 5	4 (26.7) 4	6 (40.0) 7	14 (30.4) 16	22 (24.2) 24
Myalgia	1 (6.7) 1	5 (33.3) 5	4 (26.7) 4	10 (22.2) 10	5 (31.3) 7	1 (6.7) 1	5 (33.3) 5	11 (23.9) 13	21 (23.1) 23
Nausea	2 (13.3) 3	2 (13.3) 2	3 (20.0) 3	7 (15.6) 8	1 (6.3) 2	3 (20.0) 3	2 (13.3) 2	6 (13.0) 7	13 (14.3) 15
Neutropenia	1 (6.7) 1	1 (6.7) 1	2 (13.3) 2	4 (8.9) 4	1 (6.3) 1	1 (6.7) 1	2 (13.3) 2	4 (8.7) 4	8 (8.8) 8
Abdominal pain	1 (6.7) 1	4 (26.7) 4	0	5 (11.1) 5	0	0	0	0	5 (5.5) 5
Diarrhoea	1 (6.7) 1	3 (20.0) 3	0	4 (8.9) 4	0	1 (6.7) 1	0	1 (2.2) 1	5 (5.5) 5
Injection site erythema	1 (6.7) 1	2 (13.3) 2	1 (6.7) 1	4 (8.9) 4	0	0	1 (6.7) 1	1 (2.2) 1	5 (5.5) 5

N=number of subjects in SAF, n=number of subjects with at least one AE in each category, SAF=Safety Analysis Population, #E=number of events in each category, %=n/N.

All subjects are counted only once per treatment in each AE category. Sorting order: Descending by the number of subjects in rightmost total column by PT. In case of ties, ascending order by PT Code is applied. LD= Low dose ($5x10^{10}$ viral particles), ID= Intermediate Dose ($1x10^{11}$ viral particles), HD= High Dose ($2x10^{11}$ viral particles).

Supplementary table 3: Documented SARS-CoV-2 exposures

Volunteer ID-ARM	Event type (exposure/documented infection)	Event timing (date-study w post vax)	Clinical presentation Symptoms if any/cluster description	Volunteer Clinical FU (i.e. swab date/outcome)
101013 (arm1)	Exposure (cohabitant)	20/10/2020 W8	volunteer asymptomatic<br>spouse swab positive (20/10/2020)<br>one child swab positive (26/10/2020)<br>two children negative	21/10/2020 (negative)<br>26/10/2020 (negative)<br>02/11/2020 (negative)<br>09/11/2020 (negative)
101041 (arm3)	Exposure (cohabitant)	22/10/2020 W4	volunteer asymptomatic<br>spouse swab positive (27/10/2020), child swab negative	28/10/2020 (negative)<br>03/11/2020 (negative)<br>09/11/2020 (negative)
102001 (arm1)	Seroconversion to S and N	14/09/2020 W1	volunteer completely asymptomatic for all the intercurrent period between seroconversion, its discovery and the molecular swab occurring 1 month later	14/10/2020 (negative)
102009 (arm1)	Exposure (occasional)	30/10/2020 W3	volunteer asymptomatic	02/11/2020 (negative)<br>07/11/2020 (negative)
102045 (arm3)	Exposure (occasional)	14/11/2020 W5	volunteer asymptomatic	18/11/2020 (negative)<br>24/11/2020 (negative)
102027 (arm2)	Exposure (occasional)	12/11/2020 W7	volunteer asymptomatic	17/11/2020 (negative)<br>21/11/2020 (negative)
102024 (arm2)	Exposure (occasional)	14/11/2020 W7	volunteer asymptomatic	20/11/2020 (negative)<br>25/11/2020 (negative)
102031 (arm3)	Exposure (occasional)	28/10/2020 W3	volunteer asymptomatic	02/11/2020 (negative)
102031 (arm3)	Exposure (occasional)	19/11/2020 W6	volunteer asymptomatic	24/11/2020 (negative)<br>28/11/2020 (negative)
102063 (arm4)	Exposure (cohabitant)	27/11/2020 W3	volunteer asymptomatic<br>spouse swab positive (27/11/2020)	01/12/2020 (negative)<br>04/12/2020 (negative)<br>09/12/2020 (negative)
102044 (arm3)	Documented SARS-CoV-2 infection<br>Exposure (occasional)	22/11/2020 W6	Fever, chills, headache, loss of taste and smell	27/11/2020 (positive)<br>02/12/2020 (positive)<br>09/12/2020 (undetermined)<br>14/12/2020 (undetermined)<br>18/12/2020 (negative)
102002 (arm1)	Exposure (occasional)	08/12/2020 W13	volunteer asymptomatic	14/12/2020 (negative)<br>21/12/2020 (negative)
102028 (arm2)	Exposure (occasional)	06/12/2020 W10	volunteer asymptomatic	11/12/2020 (negative)<br>16/12/2020 (negative)
102091 (arm6)	Exposure (cohabitant)	16/12/2020 W2	volunteer asymptomatic<br>son-in-law swab positive (16/12/2020)<br>child swab positive (18/12/2020)<br>spouse swab positive (19/12/2020)	19/12/2020 (negative)<br>23/12/2020 (negative)<br>28/12/2020 (negative)
102037 (amr3)	Exposure (occasional)	23/12/2020 W11	volunteer asymptomatic	28/12/2020 (negative)<br>02/01/2021 (negative)
102038 (arm3)	Exposure (occasional)	21/12/2020 W11	volunteer asymptomatic	29/12/2020 (negative)
102068 (arm5)	Exposure (occasional)	17/01/2021 W8	volunteer asymptomatic	20/01/2021 (negative)
102068 (arm5)	Exposure (cohabitant)	20/01/2021 W9	volunteer asymptomatic<br>spouse swab positive (20/01/2021)	25/01/2021 (negative)<br>29/01/2021 (negative)<br>08/02/2021 (negative)
102004 (arm1)	Exposure (occasional)	20/01/2021 W21	volunteer asymptomatic	22/01/2021 (negative)<br>29/01/2021 (negative)
102045 (arm3)	Exposure (occasional)	30/01/2021 W16	volunteer asymptomatic	05/02/2021 (negative)

102039<br>(arm2)	Exposure (occasional)	19/02/2021<br>W21	volunteer asymptomatic	24/02/2021 (negative)<br>01/03/2021 (negative)
102081<br>(arm5)	Exposure (occasional)	27/02/2021<br>W14	volunteer asymptomatic	03/03/2021 (negative)<br>08/03/2021 (negative)

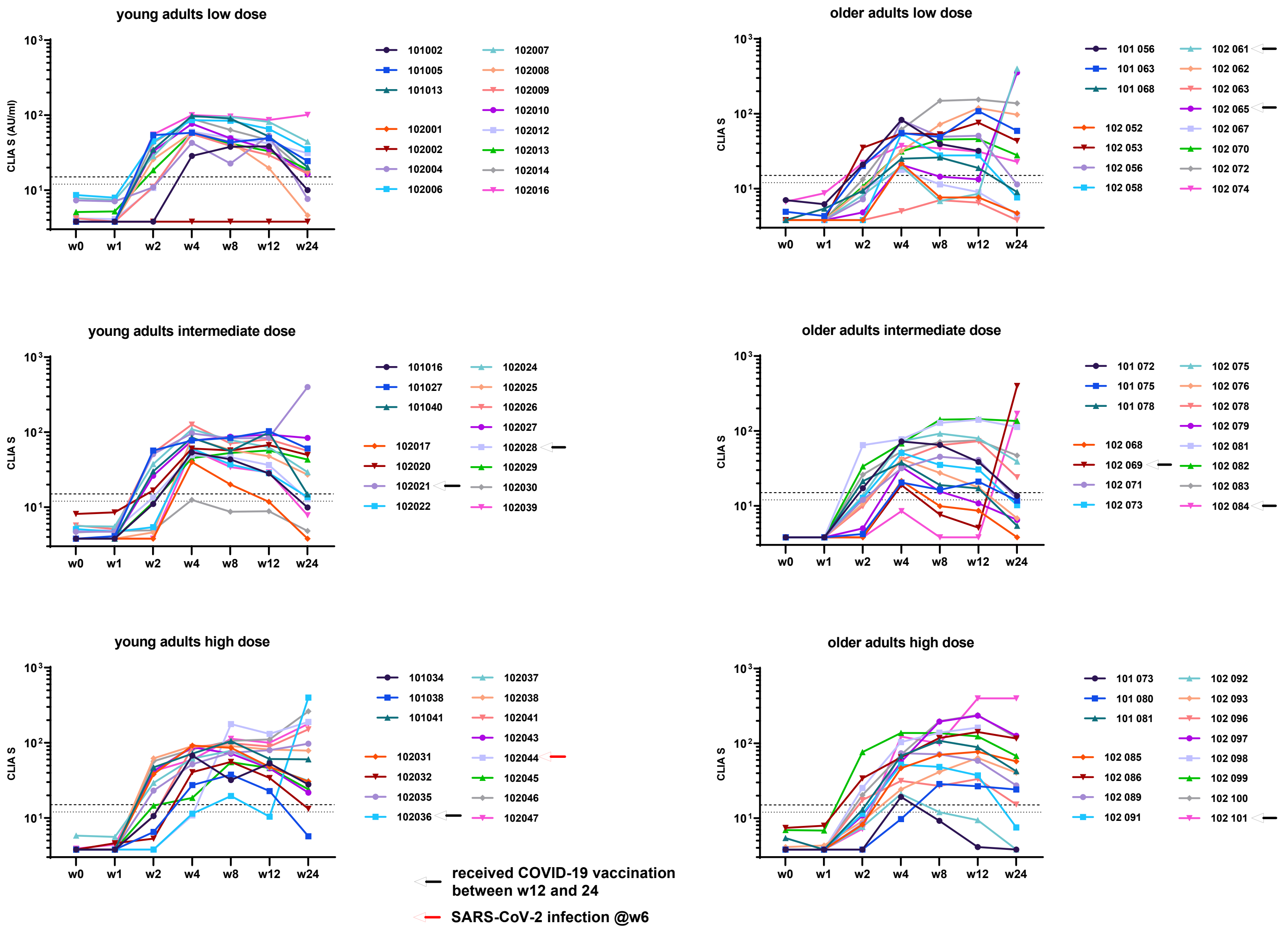

Supplementary Figure 1: CLIA kinetics in individual volunteers.

IgG binding to S1-S2 measured by CLIA at day of vaccination (w0) and 1, 2, 4, 8, 12 and 24 weeks (w1, w2, w4, w8, w12, w24 respectively) after vaccination over 6 months follow up. Data are expressed as arbitrary unit (AU)/ml. Blue and red dashed lines are set at 12 and 15 AU/ml. According to manufacturer, results >15 are clearly positive, between 12 and 15 are equivocal and <12 are negative or may indicate low level of IgG antibodies to the pathogen. In each graph, lines represent donors of young or older age cohorts at tested doses: low dose ($5x10^{10}$ vp), intermediate dose ($1x10^{11}$ vp) and high dose ($2x10^{11}$ vp). Negative samples were assigned a value of ½ the LOD. Black arrows indicate the donors which received an authorized COVID-19 vaccination between w12 and w24, red arrow indicates donor which got SARS-CoV2 infection at week 6 post vaccination.

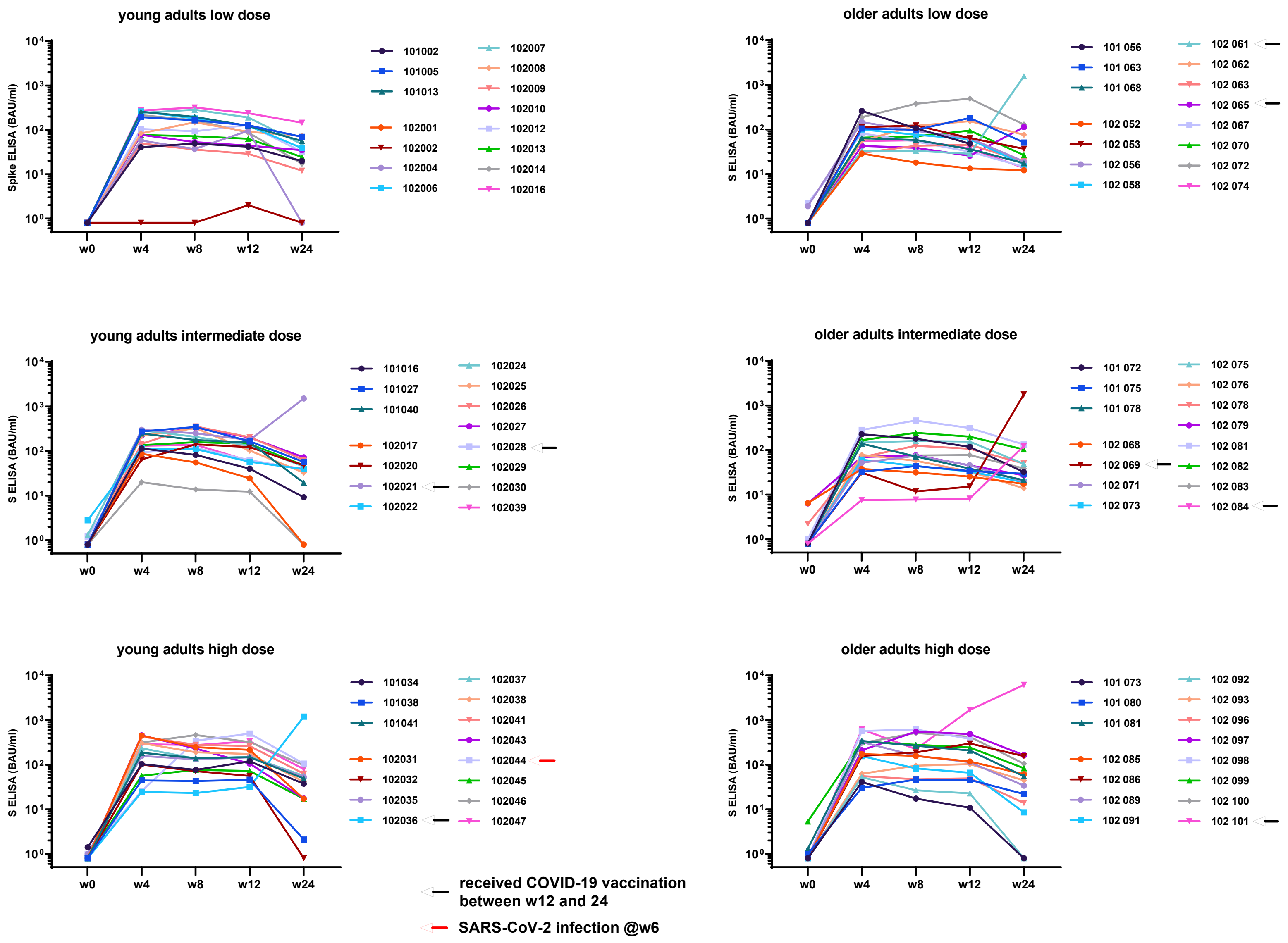

Supplementary Figure 2: Spike ELISA kinetics in individual volunteers

SARS-CoV-2–specific IgG titers in the serum collected at w0, w4, w8, w12 and w24 after vaccination measured by ELISA on recombinant full-length trimeric spike protein. Data are expressed as Binding International Unit (BAU)/mL, and, for negative serum where a titer cannot be calculated, an arbitrary value of 1 BAU/mL was assigned. Each line represents a single volunteer; arrows indicate volunteers which had either received an additional dose of a commercially-available vaccine (black arrows) or resulted positive for SARS-CoV-2 (red arrows).

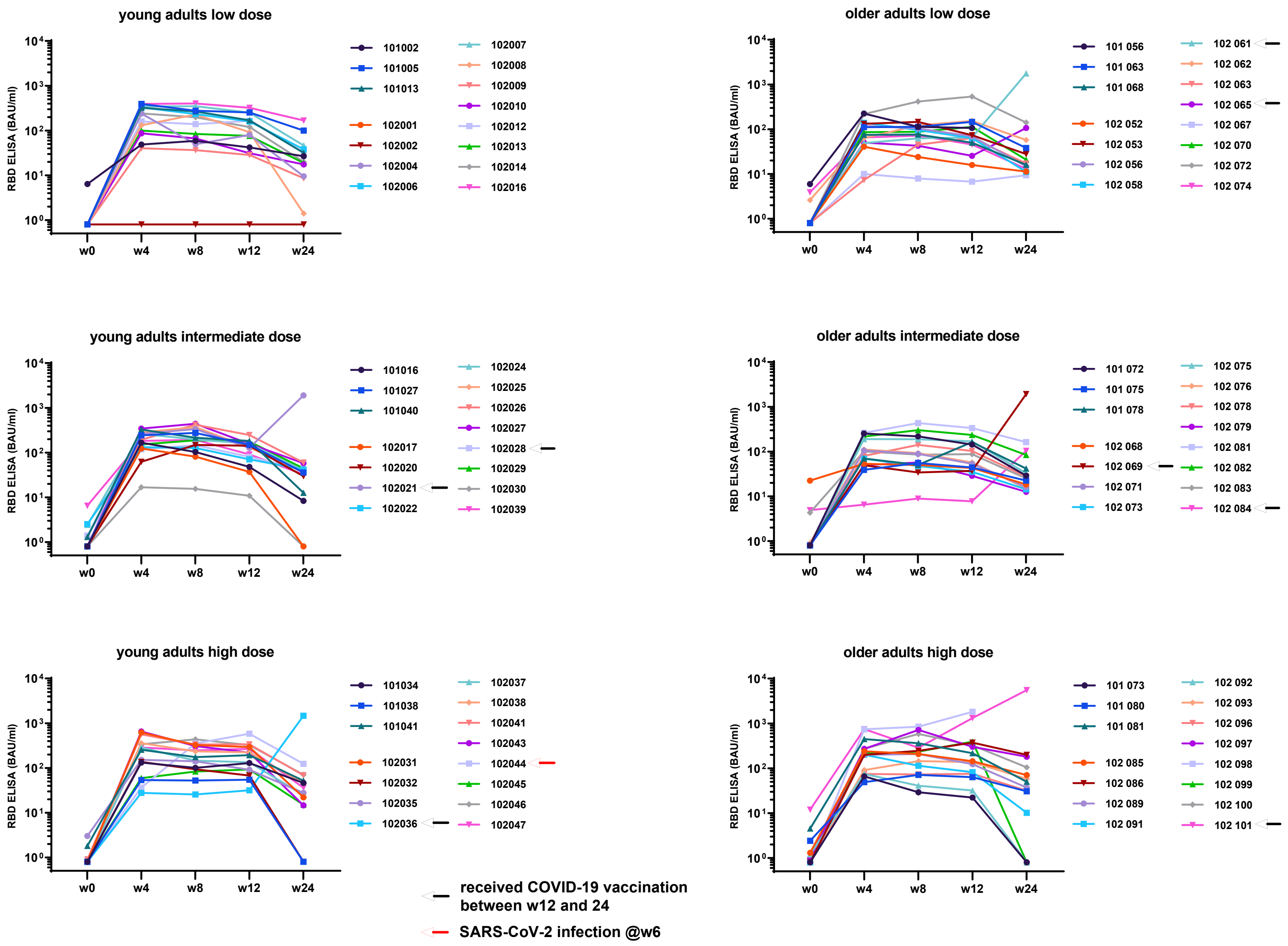

Supplementary Figure 3: RBD ELISA kinetics in individual volunteers

SARS-CoV-2–specific IgG titers in the serum collected at d0, w4, w8, w12 and w24 after vaccination measured by ELISA on recombinant RBD protein. Data are expressed as BAU/mL, and, for negative serum where a titer cannot be calculated, an arbitrary value of 1 BAU/mL was assigned. Each line represents a single volunteer; arrows indicate volunteers which had either received an additional dose of a commercially-available vaccine (black arrows) or resulted positive for SARS-CoV-2 (red arrows).

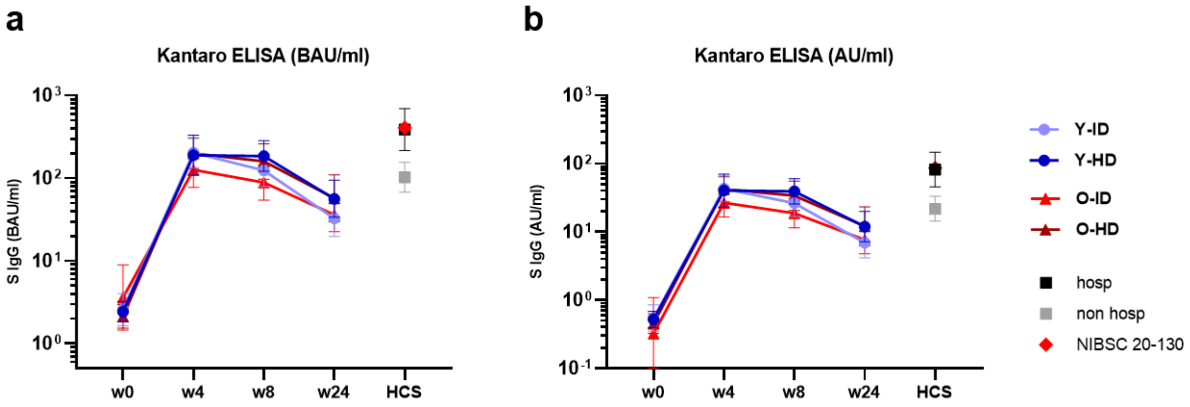

Supplementary Figure 4: Kantaro ELISA kit dataset expressed in BAU/ml and AU/ml (per manufacturer)
SARS-CoV-2–specific IgG titers in the serum collected at d0, w4, w8 and w24 after vaccination measured by Kantaro Quantitative SARS-CoV-2 IgG Antibody RUO Kit on recombinant full-length Spike protein. Data are expressed as either (a) BAU/mL or as (b) Arbitrary Units (AU)/mL. Each line represents the geometric mean value of the indicated group of volunteers, bars indicate 95% confidence interval (CI). As reference, geometric mean titers of hospitalized (black squares) and non-hospitalized (grey squares) COVID-19 patients were reported (with 95% CI). WHO research reagent plasma NIBSC 20-130 BAU/mL titer is also reported (red diamond).

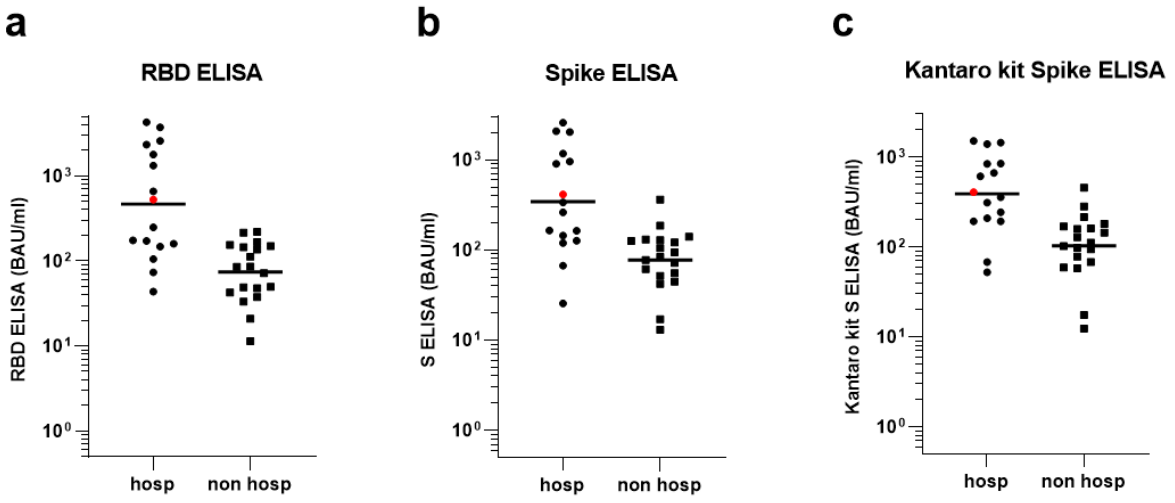

Supplementary Figure 5: ELISA in individual convalescent COVID-19 patients

SARS-CoV-2–specific IgG titers in the serum of hospitalized (circles) and non-hospitalized (squares) COVID-19 patients measured by ELISA on (a) recombinant RBD, (b) recombinant full-length Spike and (c) Kantaro Quantitative SARS-CoV-2 IgG Antibody RUO Kit. Data are expressed as BAU/mL and lines indicate geometric mean values for each group. Red circles indicate WHO research reagent plasma NIBSC 20-130.

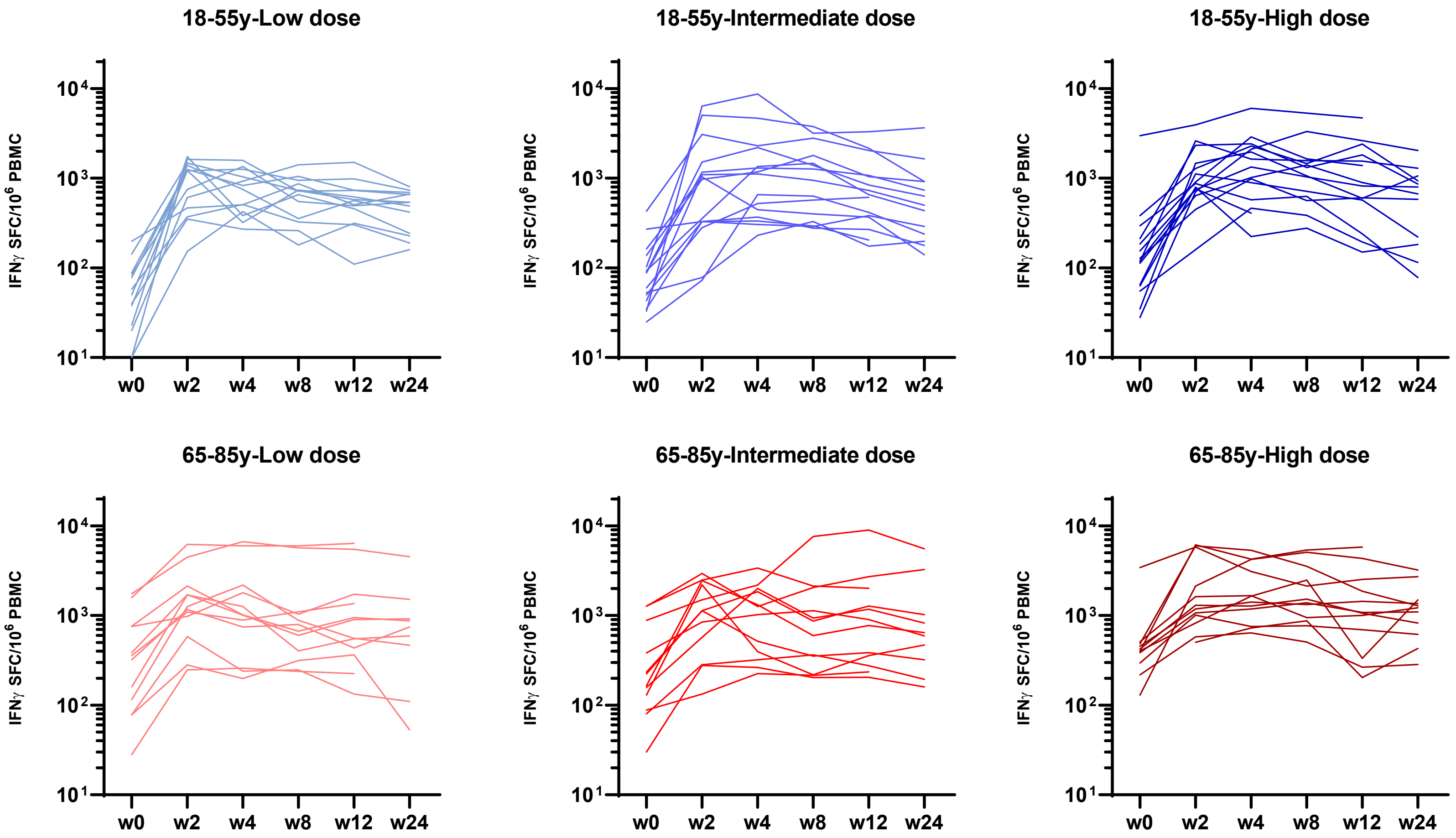

Supplementary Figure 6: SARS-CoV-2 cellular response elicited by GRAd-COV2 vaccination over time.
Spike-specific T cell response measured by IFN-γ ELISpots on frozen PBMC at day of vaccination (w0) and 2, 4, 8, 12 and 24 weeks after vaccination over 6 months follow up. Data are expressed as IFN-γ spot forming cells (SFC) per 10^6 PBMC and Spike T cell response is calculated by summing the response to each S1a, S1b, S2a and S2b peptide pools stimulation and correcting for background (DMSO stimulation) in each volunteer. Blue and red color lines identify younger (18-55 years) and older (65-85 years) age cohorts, respectively, and color tone increase with vaccine dose: low dose ($5x10^{10}$ vp), intermediate dose ($1x10^{11}$ vp) and high dose ($2x10^{11}$ vp).

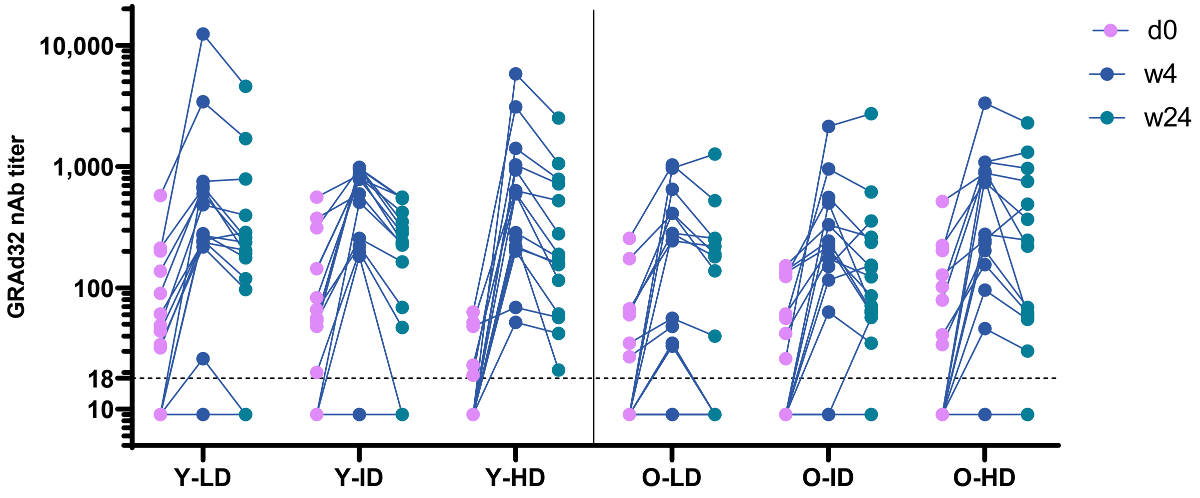

Supplementary Figure 7: GRAd neutralizing antibodies in individual volunteers

Neutralizing titers to the GRAd vector measured in serum from vaccinated volunteers the day of vaccination (d0; purple circles), 4 weeks after vaccination (w4; blue circles) and 24 weeks after vaccination (w24; green circles). The black dashed line set at 18 indicates the assay LOD;
